# (11*R*)-13-Dimethyl­ammonio-11,13-dihydro-4,5-epoxy­costunolide semifumarate

**DOI:** 10.1107/S1600536809021941

**Published:** 2009-06-13

**Authors:** Sundar Neelakantan, Sean Parkin, Peter A. Crooks

**Affiliations:** a Department of Pharmaceutical Sciences, College of Pharmacy, University of Kentucky, Lexington, KY 40536, USA; b Department of Chemistry, University of Kentucky, Lexington, KY 40506, USA

## Abstract

Crystals of the title salt, C_17_H_28_NO_3_^+^·C_4_H_3_O_4_^−^, were obtained by reacting parthenolide with dimethyl­amine followed by conversion of the amine adduct into a water-soluble fumarate salt. Subsequent crystallization of the fumarate salt from water afforded colorless ortho­rhom­bic crystals. The amine addition is highly stereospecific yielding exclusively a single diastereomer with *R*-configuration at the newly formed C-11 chiral carbon.  In the crystal, intermolecular O—H⋯O and N—H⋯O hydrogen bonds help to establish the packing.

## Related literature

Parthenolide (PTL) is a naturally occurring sesquiterpene lactone used in the treatment of fever, migraine headaches, rheumatoid arthritis, and also as an anti-inflammatory agent (Heptinstall *et al.* (1988 ▶). For the potent anti-tumor and cytotoxic properties of PTL, see: Crooks *et al.* (2007 ▶). The absolute stereochemistry of the C-11 chiral carbon is typical of such amine adducts of parthenolide, see: Nasim *et al.* (2007*a* ▶,*b* ▶). For bond-length data, see: Allen *et al.* (1987 ▶).
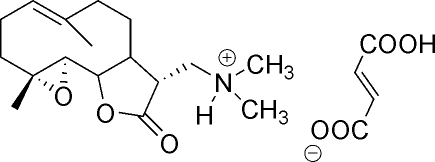


## Experimental

### 

#### Crystal data

C_17_H_28_NO_3_^+^·C_4_H_3_O_4_^−^
*M* *_r_* = 409.47
Orthorhombic, 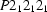

*a* = 6.3164 (1) Å
*b* = 15.1650 (2) Å
*c* = 22.0028 (3) Å
*V* = 2107.61 (5) Å^3^
*Z* = 4
Cu *K*α radiation
μ = 0.80 mm^−1^
*T* = 90 K
0.26 × 0.20 × 0.10 mm

#### Data collection

Bruker X8 Proteum diffractometer
Absorption correction: multi-scan (*SADABS* in *APEX2*; Bruker–Nonius, 2006 ▶) *T*_min_ = 0.788, *T*_max_ = 0.924
16991 measured reflections
3762 independent reflections
3640 reflections with *I* > 2σ(*I*)
*R*_int_ = 0.041

#### Refinement

*R*[*F* ^2^ > 2σ(*F* ^2^)] = 0.027
*wR*(*F* ^2^) = 0.069
*S* = 1.04
3762 reflections
268 parameters
H-atom parameters constrained
Δρ_max_ = 0.20 e Å^−3^
Δρ_min_ = −0.14 e Å^−3^
Absolute structure: Flack (1983 ▶), 1525 Friedel pairs
Flack parameter: −0.01 (4)

### 

Data collection: *APEX2* (Bruker–Nonius, 2006 ▶); cell refinement: *SAINT* (Bruker–Nonius, 2006 ▶); data reduction: *SAINT*; program(s) used to solve structure: *SHELXS97* (Sheldrick, 2008 ▶); program(s) used to refine structure: *SHELXL97* (Sheldrick, 2008 ▶); molecular graphics: *XP* in *SHELXTL* (Sheldrick, 2008 ▶); software used to prepare material for publication: *SHELXL97* and local procedures.

## Supplementary Material

Crystal structure: contains datablocks global, I. DOI: 10.1107/S1600536809021941/hg2516sup1.cif

Structure factors: contains datablocks I. DOI: 10.1107/S1600536809021941/hg2516Isup2.hkl

Additional supplementary materials:  crystallographic information; 3D view; checkCIF report

## Figures and Tables

**Table 1:** Hydrogen-bond geometry (Å, °)

*D*—H⋯*A*	*D*—H	H⋯*A*	*D*⋯*A*	*D*—H⋯*A*
N1—H1*N*⋯O6	0.93	1.83	2.7563 (14)	172
O4—H4⋯O6^i^	0.84	1.73	2.5544 (13)	169

Symmetry code: (i) 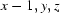.

**Fig. 1.**
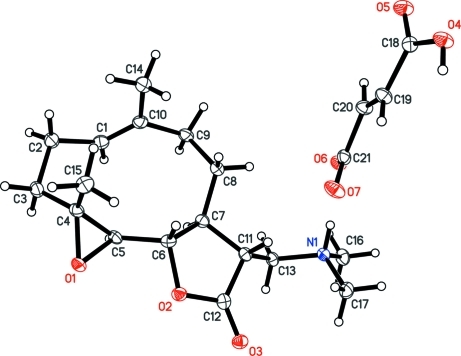
A view of the molecule (I), showing the molecule and the atom numbering scheme. Displacement ellipsoids are drawn at the 50% probability level.

**Fig. 2.**
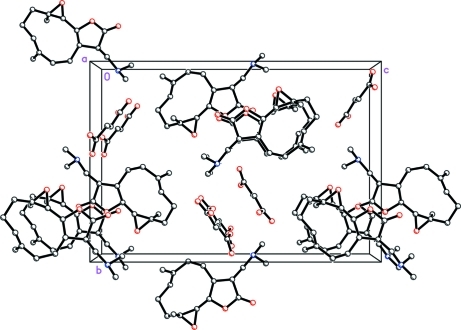
Crystal packing of (I) viewed along the a axis. H atoms have been omitted for clarity.

## supplementary crystallographic information

### Comment

Parthenolide (PTL) isolated from Tanacetum parthenium (commonly referred as feverfew), is a naturally occurring sesquiterpene lactone used in the treatment of fever, migraine headaches, rheumatoid arthritis, and also as an anti-inflammatory agent (Heptinstall *et al.*, 1988). The PTL molecule and several structurally related analogs have become topics of recent interest because of their potent anti-tumor and cytotoxic properties (Crooks *et al.*, 2007). Despite promising *in vitro* activity, this potent natural product has a major limitation which precludes its further development as a therapeutic agent, *i.e.* its poor water-solubility, thus limiting its potential as a promising clinical agent. The title compound crystallized from water as orthorombic crystals. Bond angles and bond distances within the molecule were quite regular with average normal bond lengths (Allen *et al.*, 1987). The absolute stereochemistry of the newly formed C-11 chiral carbon was found to be *R*, which is typical of such amine adducts of parthenolide (Nasim *et al.*, 2007*a*, 2007*b*). Hydrogen bonding was observed between N1—H and O6 of the carbonyl oxygen of the fumarate moiety and between O4—H and O6 (Table 1). The title compound is more water soluble and more biologically potent than the parent compound, in both *in vitro* and *in vivo* anti-leukemic activity screens. T he title compound is currently in phase 1 clinical trials.

### Experimental

The title compound (Systematic name: 13-(*N*,*N*-dimethyl)-amino-4 α,5 β- epoxy-4,10-dimethyl-6 α-hydroxy-12-oicacid-γ-lactone-germacra-1(10)-ene monofumarate) was synthesized by dissolving PTL (25 mg, 0.1 mmol) in 8 ml of methanol followed by addition of dimethylamine (0.15 mmol, 2.0*M* solution in methanol). The mixture stirred under ambient conditions for 6 hrs. The crude product was subjected to flash silica gel column chromatography to afford the pure dimethylamino analog free base. The free base was then converted to the fumarate salt by dissolving it in diethyl ether followed by addition of one equivalent of fumaric acid. The white solid that precipitated out of the diethyl ether solution was filtered, washed with diethyl ether and then dried under vacuum. Crystallization of the obtained white solid from water afforded colorless crystals that were suitable for X-ray analysis. ^1^H-NMR (D_2_0, 300 MHz):δ 6.67 2H, s, (HOOC-CH)_2_–), 5.24 (1*H*, dd, J=2.1, 12.0 Hz, 1-CH), 4.30 (1*H*, t, J=9.0 Hz, 6-CH), 1.88–3.60 (13*H*, m, 2-CH_2_, 3-CH_2_, 8-CH_2_, 9-CH_2_, 13-CH_2_, 5-CH, 7-CH, 11-CH), 2.98 (6*H*, s, N-(CH_3_)_2_), 1.70 (3*H*, s, 14-CH_3_), 1.34 (3*H*, s, 15-CH_3_) p.p.m. ^13^C-NMR (DMSO-d_6_, 75 MHz): δ 179.8 ((HOOC-CH)_2_–), 173.8 (12-C=O), 138.2 ((HOOC-CH)_2_–),137.2 (10-C), 127.3 (1-C), 85.8 (6-C), 69.2 (N-(CH_3_)2), 67.1 (5-C), 58.3 (4-C), 49.6 (7-C), 44.9 (11-C), 42.5 (9-C), 38.1 (3-C), 30.9 (8-C), 26.1(2-C), 18.7 (15-C), 18.6 (14-C)p.p.m. Elemental analysis: calc. for C_21_H_31_NO_7_: C 61.60%, H 7.63%, N 3.42%. Found: C 61.81%, H 7.64%, N 3.47%.

### Refinement

H atoms were found in difference Fourier maps and subsequently placed in idealized positions with constrained distances of 0.98 Å (RCH_3_), 0.99 Å (*R*_2_CH_2_), 1.00 Å (*R*_3_CH), 0.95 Å (C_sp2_H), 0.84 Å (O—H), 0.93 Å (N—H), and with *U*_iso_(H) values set to either 1.2*U*_eq_ or 1.5*U*_eq_ (RCH_3_, OH) of the attached atom.

### Crystal data

**Table:**

C_17_H_28_NO_3_^+^·C_4_H_3_O_4_^−^	*F*(000) = 880
*M**_r_* = 409.47	*D*_x_ = 1.290 Mg m^−^^3^
Orthorhombic, *P*2_1_2_1_2_1_	Cu *K*α radiation, λ = 1.54178 Å
Hall symbol: P 2ac 2ab	Cell parameters from 5373 reflections
*a* = 6.3164 (1) Å	θ = 3.5–68.0°
*b* = 15.1650 (2) Å	µ = 0.80 mm^−^^1^
*c* = 22.0028 (3) Å	*T* = 90 K
*V* = 2107.61 (5) Å^3^	Block, colourless
*Z* = 4	0.26 × 0.20 × 0.10 mm

### Data collection

**Table:**

Bruker X8 Proteum diffractometer	3762 independent reflections
Radiation source: fine-focus rotating anode	3640 reflections with *I* > 2σ(*I*)
graded multilayer optics	*R*_int_ = 0.041
Detector resolution: 5.6 pixels mm^-1^	θ_max_ = 68.0°, θ_min_ = 3.5°
φ and ω scans	*h* = −7→7
Absorption correction: multi-scan (*SADABS* in *APEX2*; Bruker–Nonius, 2006)	*k* = −15→18
*T*_min_ = 0.788, *T*_max_ = 0.924	*l* = −26→26
16991 measured reflections	

### Refinement

**Table:**

Refinement on *F*^2^	Hydrogen site location: inferred from neighbouring sites
Least-squares matrix: full	H-atom parameters constrained
*R*[*F*^2^ > 2σ(*F*^2^)] = 0.027	*w* = 1/[σ^2^(*F*_o_^2^) + (0.0359*P*)^2^ + 0.3552*P*] where *P* = (*F*_o_^2^ + 2*F*_c_^2^)/3
*wR*(*F*^2^) = 0.069	(Δ/σ)_max_ = 0.001
*S* = 1.04	Δρ_max_ = 0.20 e Å^−^^3^
3762 reflections	Δρ_min_ = −0.14 e Å^−^^3^
268 parameters	Extinction correction: *SHELXL97* (Sheldrick, 2008), Fc^*^=kFc[1+0.001xFc^2^λ^3^/sin(2θ)]^-1/4^
0 restraints	Extinction coefficient: 0.00093 (19)
Primary atom site location: structure-invariant direct methods	Absolute structure: Flack (1983), 1525 Friedel pairs
Secondary atom site location: difference Fourier map	Flack parameter: −0.01 (4)

### Special details

**Table:**

Geometry. All e.s.d.'s (except the e.s.d. in the dihedral angle between two l.s. planes) are estimated using the full covariance matrix. The cell e.s.d.'s are taken into account individually in the estimation of e.s.d.'s in distances, angles and torsion angles; correlations between e.s.d.'s in cell parameters are only used when they are defined by crystal symmetry. An approximate (isotropic) treatment of cell e.s.d.'s is used for estimating e.s.d.'s involving l.s. planes.
Refinement. Refinement of *F*^2^ against ALL reflections. The weighted *R*-factor *wR* and goodness of fit *S* are based on *F*^2^, conventional *R*-factors *R* are based on *F*, with *F* set to zero for negative *F*^2^. The threshold expression of *F*^2^ > 2σ(*F*^2^) is used only for calculating *R*-factors(gt) *etc*. and is not relevant to the choice of reflections for refinement. *R*-factors based on *F*^2^ are statistically about twice as large as those based on *F*, and *R*- factors based on ALL data will be even larger.Flack *x*(*u*) obtained by Parsons quotient method, as implemented in *XPREP*.

### Fractional atomic coordinates and isotropic or equivalent isotropic displacement parameters (Å^2^)

**Table:**

	*x*	*y*	*z*	*U*_iso_*/*U*_eq_	
N1	0.57684 (17)	0.48961 (7)	−0.06421 (5)	0.0150 (2)	
H1N	0.5070	0.4514	−0.0379	0.018*	
O1	0.50601 (15)	0.85986 (6)	0.14786 (4)	0.0179 (2)	
O2	0.47768 (14)	0.76214 (6)	0.03689 (4)	0.0166 (2)	
O3	0.50611 (14)	0.73366 (6)	−0.06201 (4)	0.0173 (2)	
C1	0.7548 (2)	0.65406 (9)	0.23912 (6)	0.0186 (3)	
H1	0.8861	0.6573	0.2182	0.022*	
C2	0.7092 (2)	0.72736 (9)	0.28283 (6)	0.0200 (3)	
H2A	0.8339	0.7369	0.3093	0.024*	
H2B	0.5881	0.7107	0.3090	0.024*	
C3	0.6567 (2)	0.81361 (9)	0.24889 (6)	0.0187 (3)	
H3A	0.5997	0.8572	0.2781	0.022*	
H3B	0.7881	0.8382	0.2312	0.022*	
C4	0.4967 (2)	0.79867 (9)	0.19886 (6)	0.0161 (3)	
C5	0.5848 (2)	0.77075 (8)	0.13995 (5)	0.0150 (3)	
H5	0.7427	0.7662	0.1394	0.018*	
C6	0.4759 (2)	0.71234 (9)	0.09449 (5)	0.0158 (3)	
H6	0.3272	0.6999	0.1075	0.019*	
C7	0.5921 (2)	0.62571 (8)	0.07898 (5)	0.0151 (3)	
H7	0.7481	0.6367	0.0806	0.018*	
C8	0.5420 (2)	0.54314 (9)	0.11684 (6)	0.0186 (3)	
H8A	0.5512	0.4911	0.0898	0.022*	
H8B	0.3937	0.5475	0.1311	0.022*	
C9	0.6848 (2)	0.52658 (9)	0.17255 (6)	0.0191 (3)	
H9A	0.6706	0.4642	0.1851	0.023*	
H9B	0.8341	0.5364	0.1607	0.023*	
C10	0.6331 (2)	0.58485 (9)	0.22608 (6)	0.0182 (3)	
C11	0.52671 (19)	0.61351 (9)	0.01203 (5)	0.0146 (3)	
H11	0.3850	0.5841	0.0108	0.018*	
C12	0.50272 (19)	0.70680 (9)	−0.01048 (5)	0.0152 (3)	
C13	0.6820 (2)	0.55958 (9)	−0.02661 (6)	0.0155 (3)	
H13A	0.7598	0.6001	−0.0540	0.019*	
H13B	0.7868	0.5313	0.0006	0.019*	
C14	0.4359 (2)	0.55947 (10)	0.26023 (6)	0.0238 (3)	
H14A	0.4091	0.6027	0.2924	0.036*	
H14B	0.4549	0.5009	0.2783	0.036*	
H14C	0.3154	0.5583	0.2322	0.036*	
C15	0.2737 (2)	0.77859 (10)	0.21831 (6)	0.0209 (3)	
H15A	0.2179	0.8281	0.2420	0.031*	
H15B	0.2727	0.7251	0.2433	0.031*	
H15C	0.1851	0.7695	0.1823	0.031*	
C16	0.7432 (2)	0.43816 (10)	−0.09703 (6)	0.0211 (3)	
H16A	0.6762	0.3908	−0.1204	0.032*	
H16B	0.8420	0.4127	−0.0675	0.032*	
H16C	0.8204	0.4773	−0.1247	0.032*	
C17	0.4184 (2)	0.52542 (10)	−0.10767 (6)	0.0221 (3)	
H17A	0.4871	0.5680	−0.1347	0.033*	
H17B	0.3047	0.5548	−0.0851	0.033*	
H17C	0.3589	0.4771	−0.1318	0.033*	
O4	−0.44376 (14)	0.24575 (7)	0.08148 (4)	0.0203 (2)	
H4	−0.4761	0.2890	0.0595	0.030*	
O5	−0.15889 (15)	0.19509 (7)	0.12810 (4)	0.0218 (2)	
O6	0.40728 (13)	0.37319 (6)	0.01876 (4)	0.0173 (2)	
O7	0.12629 (15)	0.46177 (7)	0.01397 (5)	0.0241 (2)	
C18	−0.2399 (2)	0.24937 (9)	0.09497 (5)	0.0161 (3)	
C19	−0.1193 (2)	0.32332 (9)	0.06681 (6)	0.0178 (3)	
H19	−0.1953	0.3724	0.0512	0.021*	
C20	0.0892 (2)	0.32310 (9)	0.06284 (6)	0.0183 (3)	
H20	0.1650	0.2766	0.0820	0.022*	
C21	0.21235 (19)	0.39254 (9)	0.02961 (6)	0.0164 (3)	

### Atomic displacement parameters (Å^2^)

**Table:**

	*U*^11^	*U*^22^	*U*^33^	*U*^12^	*U*^13^	*U*^23^
N1	0.0157 (5)	0.0132 (6)	0.0161 (5)	−0.0008 (4)	0.0006 (4)	0.0002 (4)
O1	0.0244 (5)	0.0109 (5)	0.0184 (4)	0.0016 (4)	0.0014 (4)	0.0005 (3)
O2	0.0190 (4)	0.0151 (5)	0.0159 (4)	0.0024 (4)	0.0009 (3)	0.0014 (4)
O3	0.0150 (4)	0.0191 (5)	0.0177 (4)	0.0008 (4)	−0.0013 (4)	0.0027 (4)
C1	0.0177 (6)	0.0190 (8)	0.0191 (6)	0.0032 (5)	−0.0013 (5)	0.0031 (5)
C2	0.0217 (7)	0.0186 (7)	0.0197 (6)	−0.0007 (5)	−0.0042 (5)	0.0006 (5)
C3	0.0212 (6)	0.0147 (8)	0.0201 (6)	−0.0005 (5)	0.0013 (5)	−0.0025 (5)
C4	0.0188 (6)	0.0114 (7)	0.0182 (6)	0.0029 (5)	0.0024 (5)	0.0007 (5)
C5	0.0163 (6)	0.0101 (7)	0.0184 (6)	0.0010 (5)	0.0017 (5)	0.0006 (5)
C6	0.0159 (6)	0.0162 (7)	0.0153 (5)	0.0008 (5)	0.0018 (5)	0.0026 (5)
C7	0.0147 (6)	0.0146 (7)	0.0159 (6)	−0.0003 (5)	0.0011 (5)	0.0007 (5)
C8	0.0233 (7)	0.0144 (7)	0.0181 (6)	−0.0019 (5)	0.0005 (5)	0.0000 (5)
C9	0.0247 (7)	0.0124 (7)	0.0202 (6)	0.0019 (5)	0.0004 (5)	0.0028 (5)
C10	0.0221 (7)	0.0161 (7)	0.0163 (6)	0.0036 (5)	−0.0017 (5)	0.0034 (5)
C11	0.0134 (6)	0.0136 (7)	0.0167 (6)	−0.0002 (5)	0.0009 (5)	−0.0004 (5)
C12	0.0086 (5)	0.0179 (7)	0.0192 (6)	0.0002 (5)	−0.0012 (5)	−0.0014 (5)
C13	0.0138 (6)	0.0149 (7)	0.0179 (6)	−0.0003 (5)	−0.0009 (5)	−0.0011 (5)
C14	0.0297 (8)	0.0201 (8)	0.0215 (6)	−0.0038 (6)	0.0035 (6)	−0.0005 (5)
C15	0.0181 (6)	0.0243 (8)	0.0202 (6)	0.0021 (6)	0.0026 (5)	−0.0001 (6)
C16	0.0211 (7)	0.0194 (8)	0.0227 (6)	0.0001 (6)	0.0043 (6)	−0.0050 (5)
C17	0.0232 (7)	0.0209 (8)	0.0221 (6)	−0.0002 (6)	−0.0072 (5)	−0.0012 (6)
O4	0.0131 (4)	0.0204 (5)	0.0274 (5)	−0.0009 (4)	−0.0008 (4)	0.0083 (4)
O5	0.0182 (5)	0.0228 (6)	0.0244 (5)	0.0004 (4)	0.0000 (4)	0.0074 (4)
O6	0.0126 (4)	0.0167 (5)	0.0227 (5)	−0.0007 (4)	0.0014 (4)	0.0025 (4)
O7	0.0164 (5)	0.0175 (6)	0.0383 (5)	0.0004 (4)	−0.0013 (4)	0.0075 (4)
C18	0.0140 (6)	0.0174 (7)	0.0170 (6)	0.0004 (5)	0.0014 (5)	−0.0008 (5)
C19	0.0164 (6)	0.0139 (7)	0.0230 (7)	0.0008 (5)	−0.0002 (5)	0.0018 (5)
C20	0.0160 (6)	0.0184 (8)	0.0205 (6)	0.0002 (5)	−0.0014 (5)	0.0046 (5)
C21	0.0145 (6)	0.0164 (7)	0.0183 (6)	−0.0020 (5)	−0.0027 (5)	−0.0002 (5)

### Geometric parameters (Å, °)

**Table:**

N1—C17	1.4870 (17)	C9—H9A	0.9900
N1—C16	1.4948 (17)	C9—H9B	0.9900
N1—C13	1.5005 (16)	C10—C14	1.505 (2)
N1—H1N	0.9300	C11—C12	1.5064 (19)
O1—C5	1.4507 (16)	C11—C13	1.5344 (17)
O1—C4	1.4574 (15)	C11—H11	1.0000
O2—C12	1.3474 (15)	C13—H13A	0.9900
O2—C6	1.4754 (14)	C13—H13B	0.9900
O3—C12	1.2050 (15)	C14—H14A	0.9800
C1—C10	1.332 (2)	C14—H14B	0.9800
C1—C2	1.4980 (19)	C14—H14C	0.9800
C1—H1	0.9500	C15—H15A	0.9800
C2—C3	1.5422 (19)	C15—H15B	0.9800
C2—H2A	0.9900	C15—H15C	0.9800
C2—H2B	0.9900	C16—H16A	0.9800
C3—C4	1.5114 (19)	C16—H16B	0.9800
C3—H3A	0.9900	C16—H16C	0.9800
C3—H3B	0.9900	C17—H17A	0.9800
C4—C5	1.4728 (17)	C17—H17B	0.9800
C4—C15	1.5032 (18)	C17—H17C	0.9800
C5—C6	1.5027 (18)	O4—C18	1.3224 (15)
C5—H5	1.0000	O4—H4	0.8400
C6—C7	1.5429 (18)	O5—C18	1.2128 (16)
C6—H6	1.0000	O6—C21	1.2880 (16)
C7—C8	1.5368 (18)	O7—C21	1.2314 (17)
C7—C11	1.5411 (17)	C18—C19	1.4905 (19)
C7—H7	1.0000	C19—C20	1.3199 (19)
C8—C9	1.5427 (18)	C19—H19	0.9500
C8—H8A	0.9900	C20—C21	1.4996 (18)
C8—H8B	0.9900	C20—H20	0.9500
C9—C10	1.5084 (18)		
			
C17—N1—C16	110.68 (10)	C8—C9—H9B	108.9
C17—N1—C13	113.23 (10)	H9A—C9—H9B	107.7
C16—N1—C13	108.92 (10)	C1—C10—C14	124.88 (12)
C17—N1—H1N	108.0	C1—C10—C9	120.31 (12)
C16—N1—H1N	108.0	C14—C10—C9	114.79 (12)
C13—N1—H1N	108.0	C12—C11—C13	112.50 (10)
C5—O1—C4	60.85 (8)	C12—C11—C7	103.21 (10)
C12—O2—C6	110.27 (10)	C13—C11—C7	114.98 (10)
C10—C1—C2	127.72 (12)	C12—C11—H11	108.6
C10—C1—H1	116.1	C13—C11—H11	108.6
C2—C1—H1	116.1	C7—C11—H11	108.6
C1—C2—C3	111.09 (10)	O3—C12—O2	121.29 (12)
C1—C2—H2A	109.4	O3—C12—C11	128.68 (12)
C3—C2—H2A	109.4	O2—C12—C11	110.03 (10)
C1—C2—H2B	109.4	N1—C13—C11	113.53 (10)
C3—C2—H2B	109.4	N1—C13—H13A	108.9
H2A—C2—H2B	108.0	C11—C13—H13A	108.9
C4—C3—C2	111.67 (11)	N1—C13—H13B	108.9
C4—C3—H3A	109.3	C11—C13—H13B	108.9
C2—C3—H3A	109.3	H13A—C13—H13B	107.7
C4—C3—H3B	109.3	C10—C14—H14A	109.5
C2—C3—H3B	109.3	C10—C14—H14B	109.5
H3A—C3—H3B	107.9	H14A—C14—H14B	109.5
O1—C4—C5	59.35 (8)	C10—C14—H14C	109.5
O1—C4—C15	112.71 (10)	H14A—C14—H14C	109.5
C5—C4—C15	123.12 (12)	H14B—C14—H14C	109.5
O1—C4—C3	115.99 (11)	C4—C15—H15A	109.5
C5—C4—C3	115.55 (11)	C4—C15—H15B	109.5
C15—C4—C3	116.71 (11)	H15A—C15—H15B	109.5
O1—C5—C4	59.80 (8)	C4—C15—H15C	109.5
O1—C5—C6	118.16 (10)	H15A—C15—H15C	109.5
C4—C5—C6	125.61 (11)	H15B—C15—H15C	109.5
O1—C5—H5	114.1	N1—C16—H16A	109.5
C4—C5—H5	114.1	N1—C16—H16B	109.5
C6—C5—H5	114.1	H16A—C16—H16B	109.5
O2—C6—C5	105.46 (10)	N1—C16—H16C	109.5
O2—C6—C7	104.02 (9)	H16A—C16—H16C	109.5
C5—C6—C7	115.56 (10)	H16B—C16—H16C	109.5
O2—C6—H6	110.5	N1—C17—H17A	109.5
C5—C6—H6	110.5	N1—C17—H17B	109.5
C7—C6—H6	110.5	H17A—C17—H17B	109.5
C8—C7—C11	111.41 (10)	N1—C17—H17C	109.5
C8—C7—C6	118.42 (10)	H17A—C17—H17C	109.5
C11—C7—C6	100.73 (10)	H17B—C17—H17C	109.5
C8—C7—H7	108.6	C18—O4—H4	109.5
C11—C7—H7	108.6	O5—C18—O4	121.16 (12)
C6—C7—H7	108.6	O5—C18—C19	123.02 (12)
C7—C8—C9	116.29 (11)	O4—C18—C19	115.82 (11)
C7—C8—H8A	108.2	C20—C19—C18	122.38 (13)
C9—C8—H8A	108.2	C20—C19—H19	118.8
C7—C8—H8B	108.2	C18—C19—H19	118.8
C9—C8—H8B	108.2	C19—C20—C21	123.24 (13)
H8A—C8—H8B	107.4	C19—C20—H20	118.4
C10—C9—C8	113.48 (11)	C21—C20—H20	118.4
C10—C9—H9A	108.9	O7—C21—O6	124.37 (12)
C8—C9—H9A	108.9	O7—C21—C20	120.40 (12)
C10—C9—H9B	108.9	O6—C21—C20	115.23 (12)
			
C10—C1—C2—C3	−107.32 (15)	C7—C8—C9—C10	76.93 (14)
C1—C2—C3—C4	47.74 (15)	C2—C1—C10—C14	−9.6 (2)
C5—O1—C4—C15	−116.14 (13)	C2—C1—C10—C9	168.47 (12)
C5—O1—C4—C3	105.60 (13)	C8—C9—C10—C1	−103.08 (15)
C2—C3—C4—O1	−152.71 (11)	C8—C9—C10—C14	75.20 (15)
C2—C3—C4—C5	−86.02 (14)	C8—C7—C11—C12	157.79 (10)
C2—C3—C4—C15	70.72 (15)	C6—C7—C11—C12	31.31 (11)
C4—O1—C5—C6	116.91 (12)	C8—C7—C11—C13	−79.31 (13)
C15—C4—C5—O1	98.58 (13)	C6—C7—C11—C13	154.20 (11)
C3—C4—C5—O1	−106.34 (12)	C6—O2—C12—O3	179.12 (11)
O1—C4—C5—C6	−104.76 (14)	C6—O2—C12—C11	−1.67 (13)
C15—C4—C5—C6	−6.2 (2)	C13—C11—C12—O3	34.87 (18)
C3—C4—C5—C6	148.90 (12)	C7—C11—C12—O3	159.39 (13)
C12—O2—C6—C5	144.48 (10)	C13—C11—C12—O2	−144.26 (10)
C12—O2—C6—C7	22.45 (12)	C7—C11—C12—O2	−19.74 (13)
O1—C5—C6—O2	54.19 (13)	C17—N1—C13—C11	59.61 (14)
C4—C5—C6—O2	125.63 (13)	C16—N1—C13—C11	−176.80 (10)
O1—C5—C6—C7	168.46 (10)	C12—C11—C13—N1	−109.70 (12)
C4—C5—C6—C7	−120.11 (13)	C7—C11—C13—N1	132.54 (11)
O2—C6—C7—C8	−154.30 (11)	O5—C18—C19—C20	17.7 (2)
C5—C6—C7—C8	90.61 (13)	O4—C18—C19—C20	−162.07 (13)
O2—C6—C7—C11	−32.63 (11)	C18—C19—C20—C21	174.10 (11)
C5—C6—C7—C11	−147.72 (10)	C19—C20—C21—O7	13.5 (2)
C11—C7—C8—C9	151.72 (11)	C19—C20—C21—O6	−165.65 (13)
C6—C7—C8—C9	−92.20 (14)		

### Hydrogen-bond geometry (Å, °)

**Table:**

*D*—H···*A*	*D*—H	H···*A*	*D*···*A*	*D*—H···*A*
N1—H1N···O6	0.93	1.83	2.7563 (14)	172
O4—H4···O6^i^	0.84	1.73	2.5544 (13)	169

Symmetry codes: (i) *x*−1, *y*, *z*.
